# Assessing the taxonomic status of an opecoelid trematode from the evileye blaasop, *Amblyrhynchote honckenii* (Bloch), in South Africa

**DOI:** 10.1007/s00436-026-08665-7

**Published:** 2026-05-25

**Authors:** Linda de Klerk, Russell Q-Y. Yong, Bjoern C. Schaeffner, Kerry A. Hadfield, Nico J. Smit

**Affiliations:** 1 Water Research Group, Unit for Environmental Sciences and Management, North-West University, 11 Hoffman Street, Potchefstroom, 2530 South Africa; 2 School of Veterinary Medicine, St. George’s University, True Blue, St. George’s, WI Grenada

**Keywords:** *Pseudoheterolebes cotylophorus*, *Opistholebes cf. amplicoelus*, Morphology, Molecular characterisation, Marine fish parasites, Biodiversity

## Abstract

The taxonomic status of *Pseudoheterolebes cotylophorus* (Ozaki 1935) Yamaguti 1959 (Opecoelidae: subfamily Opistholebetinae) has been a subject of taxonomic confusion since its initial description by Ozaki in 1935 as *Opistholebes cotylophorus* Ozaki 1935 from diodontid fish in Japan. The species’ current recognised classification dates back to Yamaguti’s reclassification of *O. cotylophorus* as *Pseudoheterolebes cotylophorus*; however, doubts persist regarding this classification and the validity of this species. The species was reported by Bray in 1986 from off the coast of South Africa, based on a single specimen infecting the evileye blaasop, *Amblyrhynchote honckenii* (Bloch) (Tetraodontidae). However, recently, this report from the South African coastline was considered doubtful. Parasitological assessments of South African marine fishes provided the opportunity to recollect this taxon from the same host species reported by Bray, allowing a reassessment of its taxonomic status. Morphological and molecular phylogenetic analyses, incorporating ITS2 and 28S rDNA, as well as *cox*1 and *nad*1 mtDNA sequence data, support the identification of the South African material as *Opistholebes* cf. *amplicoelus*; demonstrating both the value of molecular approaches in the identification and classification of marine parasites and the need for additional life-cycle data to fully resolve species boundaries.

## Introduction

Opistholebetine trematodes of the family Opecoelidae represent a morphologically diverse yet taxonomically complex group of marine parasites. Despite their global distribution, their true diversity remains substantially underestimated, particularly in regions such as South Africa. To date, only two of the 114 (approximately 2%) currently recognised opistholebetine species have been reported from the South African coastline (WoRMS, 2025), indicating a significant gap in our understanding of their taxonomy, biogeography, and ecological roles within marine ecosystems. This underrepresentation likely stems from limited historical sampling efforts and persistent difficulties in resolving taxonomic uncertainties (Cribb et al. 2025).

Among this limited representation, *Pseudoheterolebes cotylophorus* (Ozaki 1935); Yamaguti 1959 has been subject to taxonomic confusion, with a complex history dating back to its initial description (Martin et al. 2018a). Following its original description as *Opistholebes cotylophorus* Ozaki 1935; from the diodontid fish *Diodon holocanthus* Linnaeus in Japan (Ozaki 1935), this species has undergone multiple reassignments and misidentifications, contributing to its ambiguous status (Yamaguti 1959; Manter and Prichard 1962; Martin et al. 2018a). Yamaguti (1959) proposed the genus *Pseudoheterolebe*s Yamaguti 1959 nec Gupta 1968; based on the presence of distinctive tegument folds surrounding the ventral sucker, and designated *O. cotylophorus* as the type species of the genus, as *Pseudoheterolebes cotylophorus*. This reassignment was contested by Manter and Pritchard (1962), who prioritised internal morphological features, particularly gonad arrangement, as more reliable taxonomic traits for taxonomic classification, and reassigned the species to *Opistholebes* Nicoll 1915. Based on a single specimen, Bray (1986) reported *O. cotylophorus* from the evileye blaasop, *Amblyrhynchote honckenii* (Bloch) (Tetraodontidae), from Algoa Bay off the coast of South Africa. Later, Martin et al. (2018a) resurrected the genus *Pseudoheterolebes* to accommodate *O. cotylophorus* (now *P. cotylophorus*) along with four other species, since *O. cotylophorus* had originally been designated as the type species of the genus by Yamaguti (1959). However, they noted that Bray’s material appeared to be more consistent with species of *Opistholebes* and suggested that it represents *Opistholebes amplicoelus* Nicoll 1915; O. *microovus* Ku and Shen 1965; or potentially a new species (Martin et al. 2018a). The identity of this South African tetraodontid-infecting opistholebetine, hence, remains questionable and speculative.

These ongoing taxonomic challenges highlight the difficulty of distinguishing between closely related opistholebetine species and the limitations of traditional morphology-based taxonomy in this group (Martin et al. 2018b). These uncertainties impact our understanding of host specificity, parasite distribution, and evolutionary patterns in marine ecosystems (Cribb et al. 2025). Molecular systematics, using a multi-marker approach, incorporating ribosomal (ITS2 and 28S rDNA) and mitochondrial (*cox*1 and *nad*1 mtDNA) markers, has become a powerful tool in resolving these ambiguities and clarifying species identities and evolutionary relationships (Bray et al. 2016; Fayton and Andres 2016; Martin et al. 2018b). These methods are particularly effective in addressing the challenges posed by ambiguous or convergent morphological traits (Martin et al. 2018b). Given these challenges, the present study reassesses the identity of *P. cotylophorus sensu* Bray (1986) from *A. honckenii* off the south coast of South Africa. Information on species of *Opistholebes* and *Pseudoheterolebes*, including host records, geographical distribution, and available GenBank data, was compiled for generic comparisons (see Table 1). By refining generic classifications and integrating morphological and molecular approaches, this study aims to advance knowledge of opistholebetine systematics, highlighting the importance of combining traditional morphological methods with modern molecular tools to address species diversity and facilitate species identification of complex marine parasitic taxa.

**Table 1:** Updated information for species of *Opistholebes* Nicoll 1915 and *Pseudoheterolebe*s Yamaguti 1959 nec Gupta 1968; with information on host species, host families, distribution, and available GenBank data

Species	Host family	Host species	Distribution	ITS2	28S	*cox*1	References
Genus *Opistholebes * Nicoll 1915							
*O. amplicoelus* Nicoll 1915 Syns: *Heterolebes buckleyi* (Gupta 1968) Cribb, 2005; *H. dongshanensis* Liu 1999; *O.* *buckleyi* Gupta 1968; *O. dongshanensis* Liu 1999; *O. indicus* Gupta 1968; *O. tetraodontis* Gupta 1968	Tetraodontidae	**TH**: *Lagocephalus lunaris* (Bloch and Schneider) **OH**: *Dichotomyctere nigroviridis* (Marion de Procé); *Arothron manilensis* (Marion de Procé); *Tetractenos hamiltoni* (Richardson)	**TL**: Cleveland Bay, Australia **OL:** China; India; Moreton Bay, Australia	✓	✓	✓	Nicoll 1915; Gupta 1968; Zhukov 1977; Krishna and Sreeramulu 1993; Liu 1999; Olson et al. 2003; Martin et al. 2018a
*O. microovus* Ku and Shen 1965	Tetraodontidae	**TH**: *Takifugu niphobles* (Jordan and Snyder) **OH:** *Takifugu vermicularis* (Temminck & Schlegel)	**TL**: Chinese Taipei	-	-	-	Ku and Shen 1965; Shen and Qiu 1995; Martin et al. 2018a
Genus *Pseudoheterolebes * Yamaguti 1959 nec Gupta 1968							
*P. adcotylophorus* (Manter 1947) Martin, Ribu, Cutmore & Cribb, 2018 Syn: *O. adcotylophorus* Manter 1947	Diodontidae	**TH**: *Diodon holocanthus* Linnaeus	**TL**: Dry Tortugas, Florida, U.S.A **OL:** Cuba	-	-	-	Manter 1947; Moravec and Barus 1971; Martin et al. 2018a
*P. corazonae* Martin, Ribu, Cutmore & Cribb, 2018	Diodontidae	**TH**: *Diodon nicthemerus* Cuvier	**TL**: Stanley, Tasmania	-	-	-	Martin et al. 2018a
*P. cotylophorus* (Ozaki 1935); Yamaguti 1959 Syn: *O. cotylophorus* Ozaki 1935	Diodontidae Tetraodontidae	**TH**: *Diodon holocanthus* Linnaeus **OH:** *Chilomycterus reticulatus* (Linnaeus); *Takifugu niphobles* (Jordan and Snyder);*Trachurus mediterraneus* (Steindachner)	**TL**: Japan **OL:** Shirahama, Japan;Black Sea	-	-	-	Ozaki 1935; Ozaki 1937a, b; Yamaguti 1959; Manter and Pritchard 1962; Nikolaeva and Kovaleva 1966﻿; Yamaguti 1970; Bray 1986; Machida and Kuramochi 1999; Martin et al. 2018a
*P. diodontis* (Cable 1956) Martin, Ribu, Cutmore & Cribb, 2018 Syn: *O. diodontis* Cable 1956	Diodontidae	**TH**: *Diodon hystrix* Linnaeus **OH**:* D. liturosus* Shaw; *Lagocephalus inermis* (Temminck & Schlegel)	**TL**: Puerto Rico **OL:** Curacao; Jamaica; Hawaii, U.S.A.; Arabian Sea off Karachi, Pakistan; India; Heron Island, Australia; Lizard Island, Australia.	✓	✓	✓	Cable 1956; Siddiqi and Cable 1960; Manter and Pritchard 1962; Nahhas and Cable 1964; Bilqees 1981; Bilqees and Nighat 1982; Dyer et al. 1985, 1992; Prasad and Radhakrishnan 2010; Martin et al. 2018a
*P. stellaglobulus* Martin, Ribu, Cutmore & Cribb, 2018	Diodontidae	**TH**: *Dicotylichthys punctulatus* Kaup	**TL**: Green Island, Moreton Bay, Australia **OL:** Moreton Bay, Australia.	✓	✓	✓	Martin et al. 2018a

*OH* Other hosts, *OL* Other localities, *Syn* Synonym, *TH* Type host, *TL* Type locality

## Materials and methods

### Collection of specimens

**Fig. 1:**
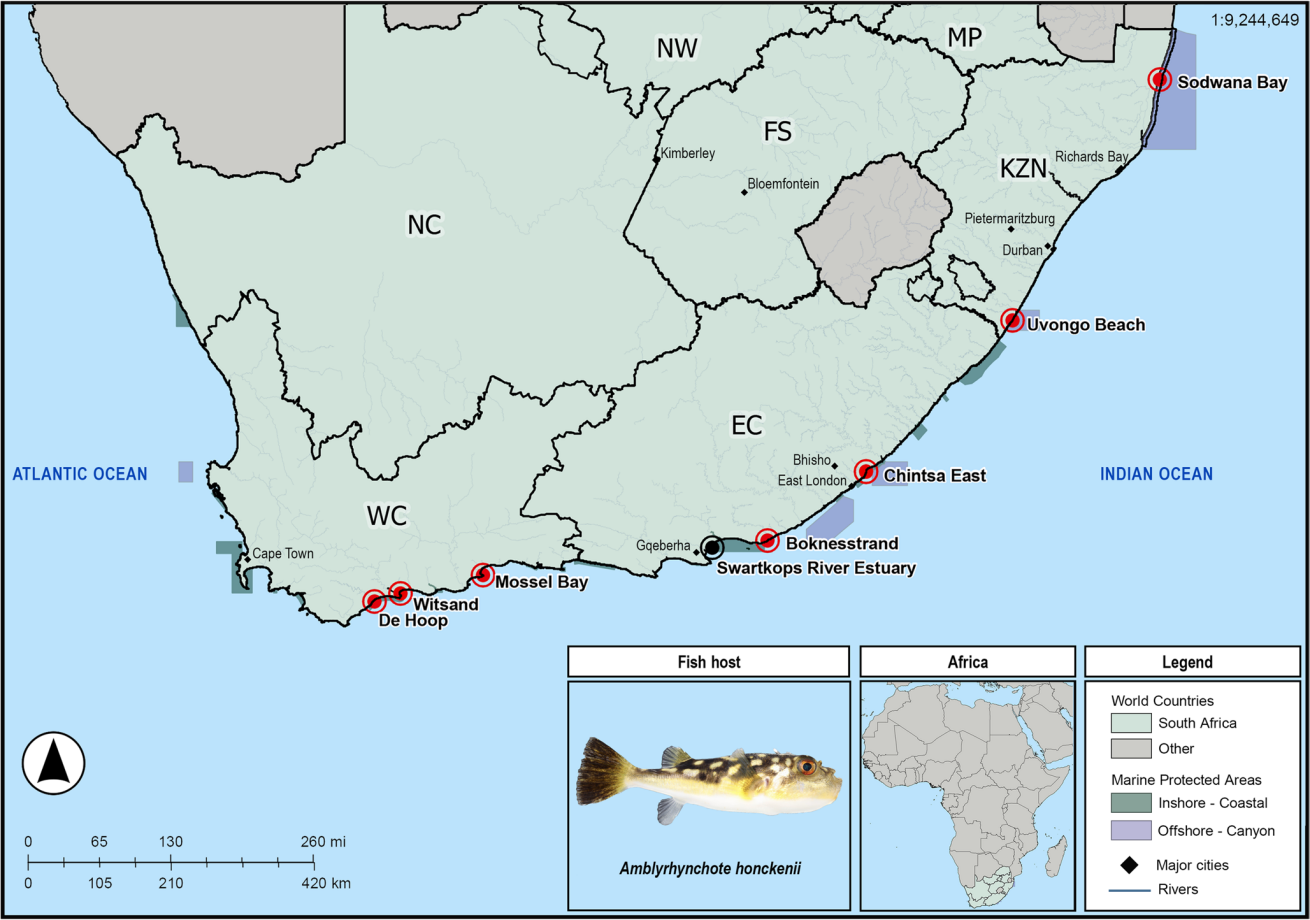
Map indicating the sampling localities of specimens of *Amblyrhynchote honckenii* (Bloch)

Seventy-four specimens of *A. honckenii* were collected, using rod and reel, from seven coastal locations along the South African coastline, arranged from west to east in Table 2. The table lists each sampling location, along with its corresponding geographic coordinates, the number of individual fish collected, and the sampling periods (see Table 2; Fig. 1). Following capture, the fish were transported in aerated containers to a nearby field station for dissection. Each specimen was identified, photographed, weighed, measured, and humanely killed by percussive stunning followed by spinal severance and pithing, according to the standard operating procedure approved by the North-West University Ethics Committee (NWU-00267-17-A5). Fish nomenclature follows FishBase (Froese and Pauly 2025).

**Table 2:** Infection parameters of *Opistholebes* cf. *amplicoelus* in *Amblyrhynchote honckenii* (Bloch) specimen collections along the South African coastline. The table details each sampling location with geographic coordinates, number of fish collected, prevalence of infection and sampling periods

Location	GPS coordinates	Prevalence %	Intensity of infection	Sampling period
De Hoop Nature Reserve, Western Cape	-34.48° S, 20.51° E	100%	8	Nov 2021
Breede River estuary at Witsand, Western Cape	-34.40° S, 20.84° E	40%	3–12	Nov 2021
Mossel Bay, Western Cape	-34.18° S, 22.15° E	0%	-	Mar 2022
Boknesstrand, Eastern Cape	-33.73° S, 26.58° E	100%	2–6	Apr 2025
Chintsa East, Eastern Cape	-32.84° S, 28.12° E	23%	1–13	Jul 2022
Uvongo Beach, KwaZulu-Natal	-30.84° S, 30.40° E	0%	-	Dec 2022
Sodwana Bay, iSimangaliso National Park, KwaZulu-Natal	-27.54° S, 32.68° E	0%	-	Sept 2022

### Morphological analyses

Internal organs of each fish were carefully removed and examined for parasites using a Zeiss Stemi 305 compact stereomicroscope (Zeiss, Oberkochen, Germany). Digenean trematodes were carefully removed with fine needles, fixed in hot saline, and preserved in 80% ethanol for further analysis. Six specimens were selected for detailed morphological examination. These specimens were rehydrated, stained with Mayer’s haematoxylin, destained with 1% hydrochloric acid, neutralised in 1% ammonia, dehydrated in a graded ethanol series, cleared in methyl salicylate, and permanently mounted on slides using Canada balsam. Observations were made using a Nikon Y-TV55 camera mounted on a Nikon ECLIPSE Ni light microscope (Nikon, Tokyo, Japan). Measurements and photographs for each species were captured using Image-Pro Express software (Nikon, Japan). Morphological characterisation of the specimens, including measurements of internal organs and body structures, followed Martin et al. (2018a). Measurements are presented in tabular form with each value shown as a range, followed by the mean in parentheses (see Table 3). All measurements are in micrometres (µm), unless stated otherwise. Detailed drawings were created using a drawing tube attached to the microscope and digitised with Adobe Photoshop v. 23.0.1.

All raw morphometric measurements of *O. amplicoelus* from South Africa and Australia were compiled into a matrix (specimens as rows, variables as columns). R package Vegan 2.7-2.7 (Oksanen et al. 2025) was used for all statistical analyses. Each variable was normalised, log-transformed (log10) and scaled (z-score standardisation; scale) to standardise scale, reduce skewness, and minimise the influence of traits with larger variances. PERMANOVA (adonis2; free permutations; No=9999) using Euclidean distance was applied to test for any significant morphological differences between species. Group separation was evaluated using a pseudo-F statistic with significance assessed via permutation (999 permutations), and results were considered significant at *p*<0.05. Additionally, PERMDISP (betadisper) (dispersion test) was run to determine if there was homogeneity of multivariate dispersion between the two groups. A non-significant PERMDISP result (*p*>0.05) indicated comparable dispersion between groups, supporting the interpretation that any significant PERMANOVA result reflected true differences in group centroids rather than unequal variability. Based on Euclidean distance, a principal component analysis (PCA; prcomp) was applied to reduce dimensionality and visualise morphological relationships in reduced multivariate space, revealing patterns of clustering or overlap between regional specimens. The PCA was visualised using the ggplot2 (Wickham 2016) package in R.

Voucher specimens were deposited in the Parasite Collection at the National Museum (NMB) in Bloemfontein, South Africa, and the Iziko South African Museum (SAM) in Cape Town, South Africa.

**Table 3:** Morphometric information for *Opistholebes* cf. *amplicoelus * from this study and from literature, and *Pseudoheterolebes cotylophorus* (Ozaki 1935) and *Opistholebes microovus* Ku and Shen 1965 from literature. Information is presented as the range, followed by the mean. All measurements are in micrometres, unless otherwise indicated

Species	*Opistholebes* cf. *amplicoelus*	*O. amplicoelus* (as *P*. *cotylophorus*)	*O. amplicoelus*	*O. amplicoelus*	*O. amplicoelus*	*O. amplicoelus* (as *O. buckleyi*)	*O. amplicoelus* (as *O. indicus*)	*O. amplicoelus* (as *O. tetraodontis*)	*O. amplicoelus* (as *Heterolebes dongshanensis*)	*P*. *cotylophorus*	*P*. *cotylophorus*	*P*. *cotylophorus*
Reference	**Present study**	Bray (1986)	Nicoll (1915) *	Martin et al. (2018a)	Zhukov (1977)	Gupta (1968)	Gupta (1968)	Gupta (1968)	Liu (1999)	Ozaki (1935) *	Ozaki (1937a, b)	Yamaguti (1959)
Host family	**Tetraodontidae**	Tetraodontidae	Tetraodontidae	Tetraodontidae	Tetraodontidae	Tetraodontidae	Tetraodontidae	Tetraodontidae	Tetraodontidae	Diodontidae	Diodontidae	Diodontidae
Location	**South Africa**	Algoa Bay, South Africa	Australia	Australia	India	India	India	India	China	Japan	Japan	Hawaii, USA
BL	**954–1**,**679 (1**,**349)**	1,220	2,000	612–1,393 (1,036)	930	735–975	1,210–1,950	890–937	960–1,600 (1,340)	2,900–3,800	800–3,800	2,100–2,800
BW	**716–1**,**330 (1**,**021)**	780	1,300	406–1,096 (761)	720	555–875	630–1,270	574–688	690–1,130 (884)	2,300–3,600	600–2,300	2,300–2,800
BL/BW	**1.06–1.52 (1.31)**	1.56	-	1.08–1.74 (1.37)	-	-	-	-	-	1.06–1.26	-	-
FBL	**689–1**,**219 (948)**	-	-	444–994 (764)	-	-	-	-	-	-	-	-
FBL/BL (%)	**58–78 (69)**	-	-	67–79 (74)	-	-	-	-	-	-	-	-
OSL	**248–482 (349)**	300	-	165–285 (220)	200	116 − 120	75–215	118–130	184–240 (210)	-	-	200–300
OSW	**223–474 (344)**	300	280	163–561 (233)	140	125–140	150–315	135–156	176–272 (227)	-	-	300–420
OSW/L	**0.78–1.22 (0.99)**	1.00	-	0.89–2.07 (1.05)	-	-	-	-	-	-	-	-
VSL	**231–458 (341)**	280	-	154–321 (238)	170	180–185	180–375	153–162	192–280 (224)	-	-	500–680
VSW	**313–564 (441)**	400	340	229–408 (290)	220	187–190	135–285	197–220	216–316 (261)	-	600–800	560–730
VSW/L	**1.09–1.53 (1.31)**	1.43	-	1.01–1.53 (1.23)	-	-	-	-	-	1.50	-	-
VSL/OSL	**0.91–1.06 (0.98)**	0.93	-	0.81–1.26 (1.08)	-	-	-	-	-	-	-	-
VSW/OSW	**1.16–1.42 (1.30)**	1.33	-	0.65–1.48 (1.28)	-	1.50	-	-	-	-	-	-
PhL	**168–302 (230)**	160	170	95–241 (155)	110	135–150	75–180	77–91	104–160 (129)	-	-	170–220
PhW	**177–315 (234)**	200	220	119–208 (155)	120	135–165	90–225	128–160	136–176 (158)	-	-	260–380
PhW/L	**0.87–1.13 (1.02)**	1.25	-	0.75–1.47 (1.02)	-	-	-	-	-	-	-	-
OSL/PhL	**1.28–1.80 (1.52)**	1.88	-	1.13–1.85 (1.44)	-	-	-	-	-	-	-	-
OSW/PhW	**1.17–1.79 (1.46)**	1.50	-	1.23–3.03 (1.50)	-	-	-	-	-	-	-	-
RTL	**170–286 (213)**	140	180	82–288 (154)	-	120–135	150–180	146–160	112–184 (152)	-	-	200–300
RTW	**162–204 (185)**	180–200	220	107–223 (157)	-	105–115	180–225	135–164	128–208 (174)	-	200–350	270–380
RTW/L	**0.71–0.96 (0.89)**	1.29–1.43	-	0.67–1.37 (1.05)	-	-	-	-	-	-	-	-
LTL	**164–274 (196)**	140	180	97–241 (152)	-	-	195–225	163–175	120–184 (150)	-	-	200–300
LTW	**139–187 (172)**	180–200	220	99–273 (157)	-	-	210–255	147–182	120–192 (165)	-	200–350	270–380
LTW/L	**0.68–0.87 (0.89)**	1.29–1.43	-	0.66–2.31 (1.06)	-	-	-	-	-	-	-	-
LTL/RTL	**0.89–1.01 (0.95)**	1.00	-	0.84–1.49 (1.01)	-	-	-	-	-	-	-	-
LTW/RTW	**0.85–1.13 (0.93)**	1.00	-	0.69–1.86 (1.01)	-	-	-	-	-	-	-	-
OvL	**108–260 (184)**	160	160	77–187 (120)	-	-	105–135	129–134	120–184 (153)	-	280–360	160–200
OvW	**122–326 (201)**	200	220	75–209 (138)	-	-	120–210	75–125	144–240 (192)	-	170–220	220–340
CSL	**200–442 (295)**	280	450	116–365 (235)	-	120–300	270–420	164–209	204–320 (258)	-	-	-
CSW	**66–141 (113)**	80	125	55–158 (108)	-	75–120	-	68–72	96–114 (108)	-	-	-
CSL/BL (%)	**19–28 (22)**	23	-	14–37 (23)	-	-	-	-	-	-	-	-
PreGP/BL (%)	**37–67 (51)**	-	-	37–67 (49)	-	-	-	-	-	-	-	-
GP-VS/BL (%)	**5–22 (19)**	-	-	19–59 (30)	-	-	-	-	-	-	-	-
Egg L**	**60–65 (63)**	73–80	65–70	51–67 (57)	58	60	60	49–61	58–68 (61)	62–64	70–77	78–86
Egg W **	**34–39 (38)**	35–42	30	27–40 (33)	29–33	30	45	21–33	31–38 (34)	40–42	47–52	46–62

*B* - body, *CS* - cirrus-sac, *FB -* forebody, *GP* - genital pore, *L* - length, *LT -* left testis, *OS -* oral sucker, *Ov* - ovary, *Ph -* pharynx, *RT* - right testis, *VS* - ventral sucker, *W -* width

* – references represent original descriptions

** – egg measurements represent the average of multiple subsamples per specimen

### Molecular analyses

Genomic DNA was extracted from a total of nine specimens, including three hologenophores *sensu* Pleijel et al. (2008) collected across four locations: De Hoop Nature Reserve (*n* = 3), the Breede River Estuary at Witsand (*n* = 2), Boknesstrand (*n* = 2), and Chintsa East (*n* = 2). Extractions were performed using the PCRBiosystems Rapid DNA Extraction Kit, following the manufacturer’s instructions with slight modifications. Specifically, 10 µL of lysis buffer and 5 µL of proteinase K-containing buffer were used, and the final extracted DNA was diluted to a volume of 300 µL. Subsequent gene amplification targeted the ITS2 and partial 28S regions of ribosomal DNA (rDNA) and the *cox*1 and *nad*1 mtDNA regions of mitochondrial DNA (mtDNA) (primers and thermocycling profiles used for amplification are listed in Table 4). Successful PCR amplifications were confirmed by electrophoresis on 1% agarose gels. Verified products were submitted to Inqaba Biotechnical Industries (Pty) Ltd. in Pretoria, South Africa, for purification and sequencing.

**Table 4:** List of primers used for DNA amplification and sequencing in this study

Gene Regions	Primers	Sequences	Sources	Thermocycling profile reference
*nad*1	NDJ11	5′-AGA TTC GTA AGG GGC CTA ATA-3′	Kostadinova et al. (2003)	Kostadinova et al. (2003)
	NDJ2a	5’-CTT CAG CCT CAG CAT AAT-3’	Kostadinova et al. (2003)	
ITS2	3S	5’-GGT ACC GGT GGA TCA CGT GGC TAG TG-3’	Morgan and Blair (1995)	Yong et al. (2016)
	ITS2.2	5’-CCT GGT TAG TTT CTT TTC CTC CGC-3’	Cribb et al. (1998)	
28S	LSU5	5’-TAG GTC GAC CCG CTG AAY TTA AGC A-3’	Littlewood (1994)	Yong et al. (2016)
	300 F (sequencing only)	5’-CAA GTA CCG TGA GGG AAA GTT G-3’	Littlewood et al. (1999)	
	ECD2 (sequencing only)	5’-CCT TGG TCC GTG TTT CAA GAC GGG-3’	Littlewood et al. (1997)	
	1500R	5’-GCT ATC CTG AGG GAA ACT TCG-3’	Snyder and Tkach (2001)	
*cox*1	Digcox1Fa	5′-ATG ATW TTY TTY TTY YTD ATG CC-3′	Wee et al. (2017)	Wee et al. (2017)
	Digcox1R	5′-TCN GGR TGH CCR AAR AAY CAA AA-3′	Wee et al. (2017)	
	DICE1F	5’-ATT AAC CCT CAC TAA ATT WCN TTR GAT CAT AAG-3’	Moszczynska et al. (2009)	Vermaak et al. (2023)
	DICE14R	5’-TAA TAC GAC TCA CTA TAC CHA CMR TAA ACA TAT GAT G-3’	Van Steenkiste et al. (2015)	

Sequences were assembled, aligned, edited, and trimmed using Geneious Prime version 2024.0.7 (Biomatters, Auckland, New Zealand). Additionally, the nucleotide Basic Local Alignment Search Tool (BLAST) was used to support identification of sequence affiliations and outgroup selections (Table 5). ITS2 rDNA, partial 28S rDNA, and *cox*1 mtDNA sequences were used to confirm the species-level identity of the sequenced specimens, as no comparable sequences were available for the *nad*1 mtDNA region.

**Table 5:** List of GenBank Opistholebetinae sequences included in the phylogenetic analyses. The taxa in bold indicate sequences generated in the present study. *Neolebouria georgiensis* Gibson, 1976 was used as an outgroup for the 28S rDNA alignment and phylogenetic analyses

Taxon	Host	Locality	Accession numbers				Sources
			ITS2	28S	*cox*1	*nad*1	
*Gaevskajatrema perezi*	Unidentified fish host	France	AJ241800	AF184255	-	-	Tkach et al. (2001)
*Heterolebes maculosus*	*Diodon hystrix*	Australia	MH933871	MH933878	-	-	Martin et al. (2018a)
*Maculifer diodontis*	*Diodon hystrix*	Australia	MH933873	MH933879	-	-	Martin et al. (2018a)
*Macvicaria bartolii*	*Diplodus annularis*	Tunisia	KR149471	KR149465	-	-	Antar et al. (2015); Rima et al. (2017)
*Macvicaria crassigula*	*Calamus bajonado*	Mexico	MF166834	KJ701237	-	-	Andres et al. (2014)
*Macvicaria dubia*	*Oblada melanura*	Tunisia	KR149488	KR149470	-	-	Antar et al. (2015)
*Macvicaria gibsoni*	*Diplodus vulgaris*	Algeria	MF166830	MF166845	-	-	Rima et al. (2017)
*Macvicaria maamouriae*	*Lithognathus mormyrus*	Tunisia	KR149474	KR149468	-	-	Antar et al. (2015)
*Macvicaria mormyri*	*Sparus aurata*; Unidentified fish host	France	MF166839	AF184256	-	-	Tkach et al. (2001); Rima et al. (2017)
*Macvicaria obovata*	*Cyclope neritea*	Spain	JQ694145	JQ694147	-	-	Born-Torrijos et al. (2012)
*Magnaosimum brooksae*	*Tripodichthys angustifrons*	Australia	MG813905	MG813907	-	-	Martin et al. (2018b)
*Opistholebes amplicoelus*	*Lagocephalus lunaris*;* Tetractenos hamiltoni*	Australia	MH933868	MH933875	MH933883–MH933888	-	Martin et al. (2018a)
*Opistholebes* cf.* amplicoelus*	***Amblyrhynchote honckenii***	**South Africa**	**PX945812; PX957014**	**PX945837– PX945838**	**PX946181;** **PX946220;** **PX946035**	**PZ195469–** **PZ195470**	**Present study**
*Parallelolebes virilis*	*Meuschenia hippocrepis*	Australia	MH933874	MH933880	-	-	Martin et al. (2018a)
*Peracreadium idoneum*	*Anarhichas lupus*	UK	-	AY222209	-	-	Olsen et al. (2003)
*Propycnadenoides philippinensis*	*Gymnocranius grandoculis*	Australia	-	KU320604	-	-	Bray et al. (2016)
*Pseudoheterolebes diodontis*	*Diodon liturosus*;* Diodon hysterix*	Australia	MH933869	MH933876	-	-	Martin et al. (2018a)
*Pseudoheterolebes stellaglobulus*	*Dicotylichthys punctulatus*	Australia	MH933870	MH933877	-	-	Martin et al. (2018a)
*Pseudopycnadena fischthali*	*Diplodus vulgaris*	Algeria	MF166841	MF166851	-	-	Rima et al. (2017)
*Neolebouria georgiensis*	*Parachaenichthys charcoti*	Ukraine	-	ON123036	-	-	Faltýnková et al. (2022)

Partial 28S rDNA sequences were aligned with those of Opistholebetinae species for which sequence data were available on GenBank. *Neolebouria georgiensis* Gibson, 1976, was used as the outgroup taxon for the 28S rDNA phylogenetic analyses. Alignments were constructed, generated, and trimmed using default parameters of MAFFT v 7.490 (Katoh et al. 2002; Katoh and Stanley 2013), on the Geneious Prime platform v 2024.0.7 (Kearse et al. 2012). The *cox*1 mtDNA dataset was translated and inspected in Mesquite v 3.03 (Maddison and Maddison 2017) for stop codons per Huston et al. (2021), tested for non-stationarity caused by base composition bias using the χ^2^ test function on PAUP* (Swofford 2002), and for substitution saturation using Xia’s test function on DAMBE 7 (Xia 2018). No base composition bias was detected on any codon positions (*P*=1.00 for all codon positions), nor any substitution saturation (I_ss_=0.0318; I_ss.c_=0.7099). Genetic differences across all sequenced specimens and DNA regions were calculated on the Geneious Prime platform, with results shown as percentage similarities and number of base-pair differences.

The optimal nucleotide substitution model for each dataset was estimated using the Akaike Information Criterion (AIC) in jModelTest v 2.1.4 (Posada 2008; Darriba et al. 2012). The general time-reversible model with invariant sites and gamma-distributed rate variation (GTR+I+G) was estimated for the 28S rDNA and *cox*1 mtDNA datasets. Phylogenetic analyses were performed using Maximum Likelihood (ML) and Bayesian Inference (BI) methods with this suggested model. BI analyses were conducted on the CIPRES Science Gateway v 3.3 (Miller et al. 2010) using MrBayes v 3.2.7a (Ronquist et al. 2012), with 2 independent Markov Chain Monte Carlo (MCMC) runs of 4 chains for 10 million generations, sampling every 1,000 generations, and a burn-in of the first 2,500 generations. ML analyses were performed using PhyML version 3.0 (Guindon et al. 2010) on the ATGC bioinformatics platform, with model parameters estimated and 1,000 bootstrap repetitions for nodal support. The resulting phylogenetic trees from BI and ML analyses were visualised using TreeViewer v 2.2.0 (Bianchini and Sánchez-Baracaldo 2024).

## Results

Specimens of opistholebetine trematodes from *A. honckenii* were recovered from four localities: De Hoop Nature Reserve, the Breede River Estuary at Witsand, Boknesstrand, and Chintsa East (Table 2). Of these, eleven individuals were sexually mature and gravid, while the remainder were immature and therefore not considered for the morphological dataset. Assessments of fish from off Mossel Bay, Uvongo Beach, and Sodwana Bay did not yield any specimens of opistholebetine trematodes (Table 2). Morphological measurements of the newly collected specimens are presented in Table 3 along with published metrical data for all valid species of *Opistholebes* and *Pseudoheterolebes*. A combination of morphometric and molecular phylogenetic comparisons supported the identity of these specimens as *Opistholebes* cf. *amplicoelus*.

### Taxonomic summary

**Fig. 2:**
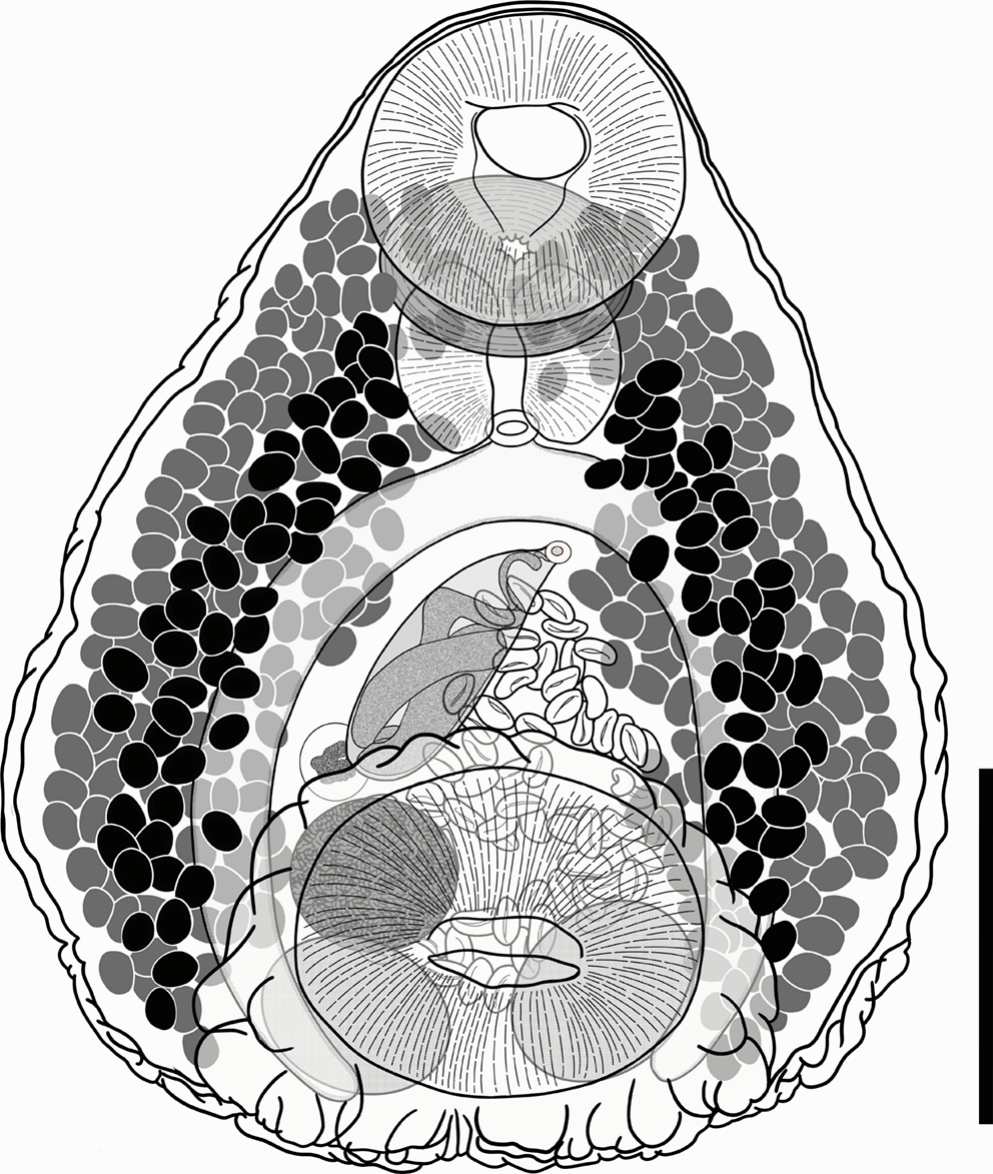
Illustration of an adult voucher specimen (NMB P-1315) of *Opistholebes* cf. *amplicoelus* from the evileye blaasop, *Amblyrhynchote honckenii* (Bloch), from Chintsa East, South Africa. Scale bar: 500 μm

*Opistholebes *cf.* amplicoelus* (Fig. 2).

#### Host

*Amblyrhynchote honckenii* (Bloch) (Tetraodontiformes: Tetraodontidae).

#### Locality

De Hoop Nature Reserve, Western Cape, South Africa (-34.48°S, 20.51°E); Breede River Estuary, Witsand, Western Cape, South Africa (-34.40°S, 20.84°E); Boknesstrand, Eastern Cape, South Africa (-33.73°S, 26.58°E); Chintsa East, Eastern Cape, South Africa (-32.84°S, 28.12°E).

#### Material studied

Morphological voucher specimens ex *A. honckenii* collected from De Hoop Nature Reserve (hologenophore SAMC-A100002); Breede River Estuary, Witsand (SAMC-A100003; NMB P-1313 and NMB P-1317, plus one hologenophore NMB P-1304); Boknesstrand (NMB P-1308; NMB P-1312; NMB P-1316 and NMB P-1320) and Chintsa East (NMB P-1035–NMB P-1307; NMB P-1309–NMB P-1311; NMB P-1314; NMB P-1318–NMB P-1319, plus one hologenophore NMB P-1315).

#### Site in host

Intestine.

#### Representative DNA sequences: ITS2 rDNA

Three identical replicates of 428 bp (one each from De Hoop Nature Reserve, Chintsa East and Boknesstrand), two submitted to GenBank (PX945812 andPX957014); 28S rDNA: Three identical replicates of 1,250–1,268 bp (one from De Hoop Nature Reserve, and two from Chintsa East), two submitted to GenBank (PX945837–PX945838); *cox*1 mtDNA: eight sequences of 475–790 bp comprising three haplotypes (two each from De Hoop Nature Reserve, Witsand, Chintsa East, and Boknesstrand), three submitted to GenBank (PX946181, PX946220 and PX946035); *nad*1 mtDNA: six sequences of 474 bp comprising two haplotypes (two each from De Hoop Nature Reserve and Chintsa East, and one each from Witsand and Boknesstrand), two submitted to GenBank (PZ195469–PZ195470).

### Description

Based on nine ventrally whole-mounted specimens and three hologenophores, measurements in Table 3. Body robustly pyriform, with rounded to flat posterior. Tegument thick, smooth to wrinkled, with variably rugose frill posteriorly. Forebody approximately three-quarters of total body length. Oral sucker subterminal, spherical to subspherical. Ventral sucker subterminal from posterior extremity, subspherical to transversely-ellipsoidal, slightly larger than oral sucker, surrounded by tegumental folds forming posterior rugose “frill”. Tegumental folds varying from almost non-existent in some specimens to somewhat extensive, but never completely enveloping ventral sucker. Pre-pharynx surrounded by prominent, muscular post-oral ring. Pharynx muscular, ellipsoidal, smaller than oral sucker. Oesophagus short. Intestinal bifurcation broad, in anterior third of body. Caeca broad, with thick internal gastrodermis, slightly tapered posteriorly, extending to level of posterior margin of ventral sucker. Testes two, spherical to ovoid, approximately equal in size, margins not contiguous, situated dorsal to ventral sucker, spanning entire intercaecal space. Cirrus-sac elongated, oval, medial. Seminal vesicle broad, tubular, looped, occupies majority of cirrus-sac, sharply constricted prior to *pars prostatica*. *Pars prostatica* short, meets genital atrium. Common genital atrium short, simple, immediately preceding common genital pore. Genital pore medial to just sinistral of midline, overlapping caeca in some specimens, entirely post-caecal in others. Ovary roughly spherical, margins smooth, slightly smaller than testes, dextro-submedial, antero-dorsal to and slightly overlapping dextral testis. Canalicular seminal receptacle prominent, smaller than and immediately anterior to ovary. Laurer’s canal short, opens dorsal to ovarian complex. Oötype not observed; Mehlis’ gland not prominent. Uterus convoluted, thin-walled, mostly pre-testicular in sinistral intercaecal region, dorsally slightly overlapping anterior margin of sinistral testis. Eggs oval, tanned, operculate. Vitelline follicles numerous, dense, relatively large, distributed from level of pharynx to posterior extremity, more extensive dorsally than ventrally; dorsal distribution fills most of body, somewhat but not entirely overlapping intercaecal space; ventral distribution partially overlapping caeca, not confluent. Pigment granules sparsely interspersed among vitelline follicles. Excretory vesicle difficult to observe, short, saccular, at most reaching the posterior margin of ovary. Excretory pore subterminal, dorsal to ventral sucker.

### Morphological remarks

This study represents the first record of the opistholebetine trematode infecting *A. honckenii* off the coast of South Africa since Bray (1986) reported a single specimen from the Swartkops River estuary, Algoa Bay, near Gqeberha. Morphological comparisons between the specimen described by Bray (1986) and those examined in the present study indicate that they belong to the same taxon. Martin et al. (2018a) previously suggested that the South African specimen identified by Bray (1986) may have closer affinities to species of *Opistholebes* than to *Pseudoheterolebes*, noting its morphological similarity to *O. amplicoelus* and *O. microovus* and proposing that it might represent a distinct, undescribed species. However, the South African specimens can be distinguished from *O. microovus* by a combination of morphological features, including a narrower ventral sucker (<564 vs. 578–957 μm in width), a shorter (<302 vs. 334–434 μm in length) and thinner pharynx (<315 vs. 418–501 μm in width), narrower testes (<273 vs. 284–367 μm in width), and a longer cirrus-sac (>116 vs. 83 μm in length) (Table 3). Comparison of these features with both the original description of *O. amplicoelus* by Nicoll (1915) and the redescription by Martin et al. (2018a) supports the identification of the South African specimens as *Opistholebes* cf. *amplicoelus* and suggests that Bray’s (1986) original classification of the specimen as *Pseudoheterolebes cotylophorus* was incorrect.

Detailed morphological comparisons among the present specimens, Bray’s (1986) specimen, and previously reported descriptions of both *O. amplicoelus* and *P. cotylophorus* (summarised in Table 3) reveal strong similarity to *O. amplicoelus* and clear distinctions from *P. cotylophorus*. The South African specimens conform to the generic characteristics of *Opistholebes* as outlined by Martin et al. (2018a), including a broad body (Fig. 2), with total length less than twice the width, and a ventral sucker positioned posteriorly, dorsally to the testes (Fig. 2), and measuring less than 1.5 times the width of the oral sucker (Table 3). These traits closely match those described by Nicoll (1915) and subsequent redescriptions (e.g. Manter and Pritchard 1962; Martin et al. 2018a), with only minor metrical variations (Table 3). Previous reports consistently note that gravid individuals of *O. amplicoelus* are smaller than those of *P. cotylophorus*, with Nicoll (1915) reporting a maximum size of 2,000 μm for *O. amplicoelus*, although the specimen was noted as flattened. In contrast, *P. cotylophorus* specimens are generally larger, ranging from 2,000 to 3,800 μm. Ozaki (1937a) reported specimens as small as 800 μm, though the maturity of these individuals was not specified. Although egg size has been considered a potential diagnostic character in opistholebetines, it does not reliably distinguish *O. amplicoelus* from *P. cotylophorus*. The egg sizes reported for *O. amplicoelus* from the type locality range from 51 to 70 × 27–40 μm (Nicoll 1915; Martin et al. 2018a), which is consistent with the present South African specimens (60–65 × 34–39 μm). However, Bray’s (1986) specimen exhibited distinctly larger eggs (73–80 × 35–42 μm), overlapping with those reported for *P. cotylophorus* as 70–77 × 47–52 μm (Ozaki 1937a) and 78–85 × 46–62 μm (Yamaguti 1959). Notably, the original description of *P. cotylophorus* by Ozaki (1935) reported egg dimensions (62–64 × 40–42 μm) that match the ones reported for *O. amplicoelus*. These findings suggest that egg size is not a reliable taxonomic character for distinguishing between these two species. A key morphological feature of *Pseudoheterolebes*, including *P. cotylophorus*, is the presence of pronounced tegumental folds or “frills” surrounding the ventral sucker. This character was a primary justification for the establishment of the genus and the designation of *P. cotylophorus* as its type species (Yamaguti 1959). However, these prominent tegumental folds are absent in Bray’s (1986) specimen and mostly absent in the South African specimens examined in the present study (Fig. 2). While some individuals exhibit slight frilling around the ventral sucker, none show the extensive development typical of *Pseudoheterolebes* species.

**Fig. 3:**
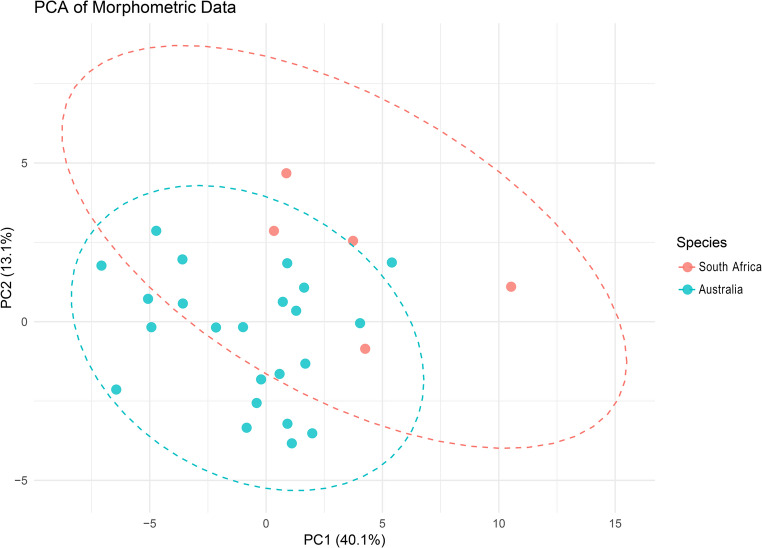
Principal Component Analysis (PCA) plot based on log-transformed and normalised morphometric measurements of *Opistholebes* cf. *amplicoelus* and *Opistholebes amplicoelus* Nicoll 1915; specimens from South Africa and Australia, respectively. Each point represents an individual specimen, with clustering reflecting morphometric similarity

The South African specimens do, however, show some marginal differences to those of Martin et al. (2018a), namely in having larger maximal sizes for forebody length, ventral sucker length and breadth, pharynx length and breadth, ovary length, and egg length and breadth. However, these differences are interpreted as being consistent with intraspecific variation. Furthermore, all these features are still within the ranges for *O. amplicoelus* when one accounts for the original description by Nicoll (1915) and the specimen observed by Bray (1986) (see Table 3). Statistical morphometric analysis further supports this interpretation by demonstrating statistically significant but incomplete separation between South African and Australian specimens of *O. amplicoelus*. PERMANOVA analysis comparing Australian and South African datasets revealed a significant difference in overall morphometric profiles between regions (pseudo-F=4.24, *p*=0.002), indicating that the two populations differ in multivariate space. However, the test produced a moderate R² value (R²=0.14016), signifying only partial separation rather than complete divergence. Importantly, PERMDISP showed no significant difference in within-group dispersion (*p*=0.525), confirming that the observed PERMANOVA result reflects differences in group centroids rather than unequal variability. Collectively, these results indicate that although the two regional populations exhibit measurable morphometric differentiation, there remains substantial overlap in morphospace (Fig. 3), consistent with intraspecific variation rather than the presence of distinct taxa.

### Molecular results

**Fig. 4:**
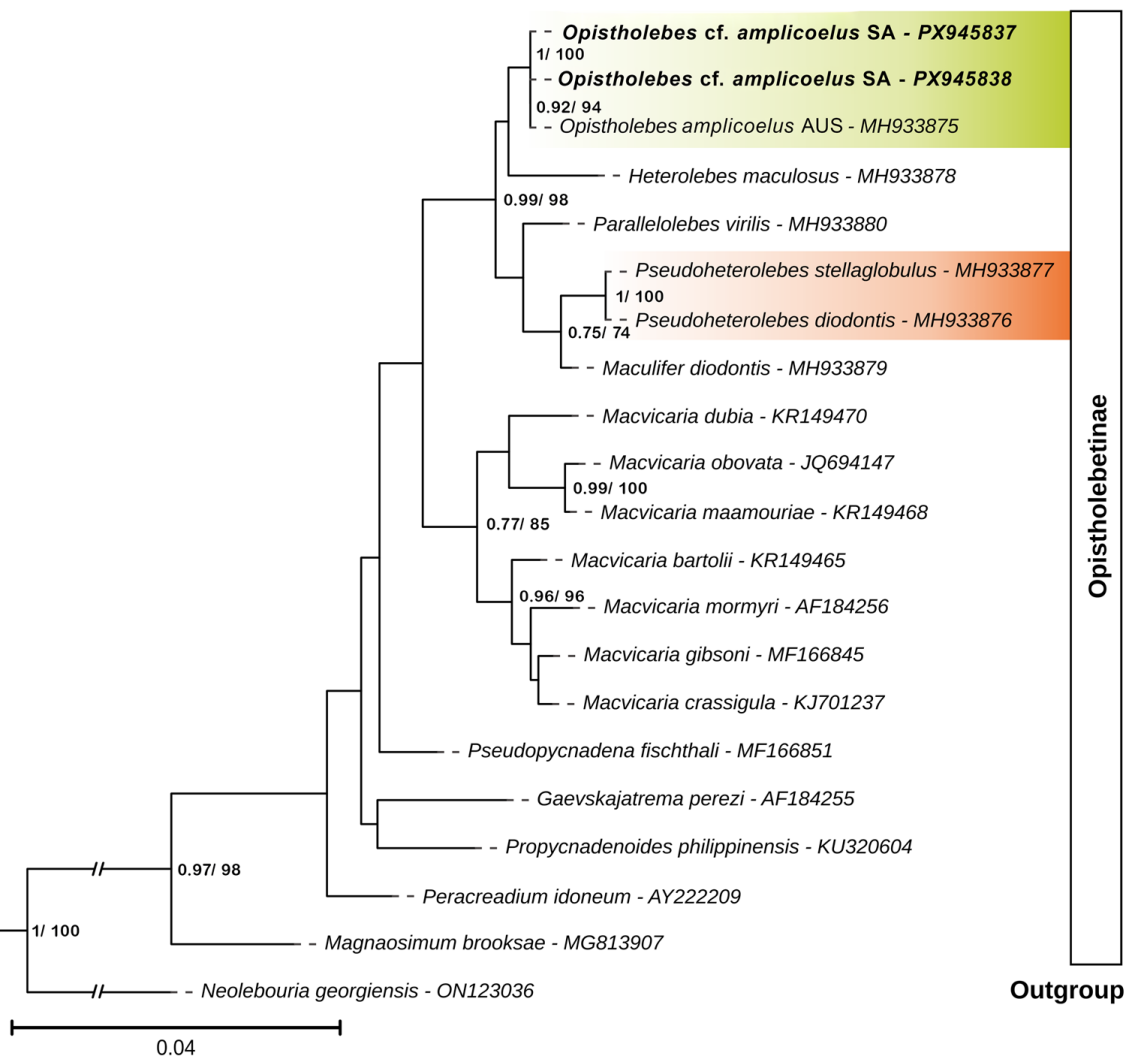
Phylogenetic tree of the subfamily Opistholebetinae based on partial 28S rRNA gene alignments. Newly generated sequences for *Opistholebes* cf. *amplicoelus*; are provided in bold. Nodal support presented above or below branches for Bayesian Inference (> 0.7) and Maximum Likelihood (> 70%) analyses (BI/ML). Dashes indicate values below 0.70 and 70%, respectively. *Neolebouria georgiensis* Gibson, 1976, was used as the outgroup. *AUS* - Australia, *SA* - South Africa

The ITS2 rDNA and partial 28S rDNA molecular phylogenetic analyses produced final alignments of 433 bp and 1,250 bp, respectively. The newly generated ITS2 and partial 28S rDNA sequences for South African specimens were identical across all specimens, exhibiting no intraspecific variation (i.e. 0 bp difference). In the ITS2 rDNA analyses, all South African sequence replicates (collected from De Hoop, Boknesstrand and Chintsa East) were identical to that of a sequence of *Opistholebes amplicoelus* from off North Stradbroke Island, Australia (ex *L. lunaris*) (Table 6). Similarly, in the partial 28S rDNA analysis, the newly generated South African specimens (collected from De Hoop and Chintsa East) showed no differences (0 bp) to the sequence of *O. amplicoelus* from off North Stradbroke Island, Australia (ex *L. lunaris*) (Table 7). Maximum likelihood (ML) and Bayesian inference (BI) analyses based on the rDNA alignment, which included partial 28S sequences of Opistholebetinae, yielded phylogenetic trees with congruent topologies and comparable nodal support (Fig. 4). These results align with previously reported phylogenetic patterns for Opistholebetinae (Martin et al. 2018a). The South African sequences formed a well-supported clade with *O. amplicoelus* (highlighted in green in Fig. 4), clearly separated from the two *Pseudoheterolebes* species, which grouped into a distinct clade (highlighted in orange). The newly generated sequences of the South African specimens showed clear genetic distinction from species of *Pseudoheterolebes*, exhibiting 17 bp differences in the 28S rDNA region (Table 7). The smallest interspecific differences (14 bp/1.06%) were observed between *O. amplicoelus* and *Maculifer diodontis* Martin, Ribu, Cutmore & Cribb, 2018. Conversely, *Magnaosimum brooksae* Martin, Crouch, Cutmore & Cribb, 2018, was the most genetically distant species from *O. amplicoelus*, with 50 bp differences (4%) in the 28S rDNA region (Table 7).

**Table 6:** Nucleotide comparison of partial ITS2 rDNA sequences of Opistholebetinae based on a 433 bp alignment

	Number of bases/residues that are not identical																		
	*Opistholebes* cf. *amplicoelus * DH	*Opistholebes* cf. *amplicoelus * BS	*Opistholebes* cf. *amplicoelus* CE	*Opistholebes amplicoelus*	*Maculifer. diodontis*	*Parallelolebes. virilis*	*Pseudoheterolebes diodontis*	*Pseudoheterolebes stellaglobulus*	*Heterolebes maculosus*	*Gaevskajatrema perezi*	*Macvicaria* *mormyri*	*Macvicaria maamouriae*	*Macvicaria* *gibsoni*	*Macvicaria* *dubia*	*Macvicaria* *bartolii*	*Macvicaria* *crassigula*	*Macvicaria* *obovata*	*Pseudopycnadena fischthali*	*Magnaosimum brooksae*
*Opistholebes* cf. *amplicoelus* DH		0	0	0	12	13	13	15	17	17	18	20	20	21	21	21	22	24	42
*Opistholebes* cf. *amplicoelus* BS	100		0	0	12	13	13	15	17	17	18	20	20	21	21	21	22	24	42
*Opistholebes* cf. *amplicoelus *CE	100	100		0	12	13	13	15	17	17	18	20	20	21	21	21	22	24	42
*Opistholebes amplicoelus*	100	100	100		12	13	13	15	17	17	18	20	20	21	21	21	22	24	42
*Maculifer diodontis*	97.21	97.21	97.21	97.21		10	10	11	14	15	17	21	20	22	20	21	23	22	38
*Parallelolebes virilis*	96.97	96.97	96.97	96.97	97.67		11	12	10	10	14	19	16	20	15	14	21	18	38
*Pseudoheterolebes diodontis*	96.98	96.98	96.98	96.98	97.67	97.44		2	18	18	15	21	18	22	18	19	23	23	40
*Pseudoheterolebes stellaglobulus*	96.51	96.51	96.51	96.51	97.44	97.2	99.53		19	19	15	21	18	22	18	19	23	22	39
*Heterolebes maculosus*	96.03	96.03	96.03	96.03	96.74	97.66	95.8	95.57		17	20	24	20	25	22	22	26	24	40
*Gaevskajatrema perezi*	96.04	96.04	96.04	96.04	96.5	97.66	95.8	95.57	96.03		12	17	14	17	13	14	19	15	36
*Macvicaria mormyri*	95.8	95.8	95.8	95.8	96.04	96.73	96.5	96.5	95.33	97.2		12	5	12	5	6	14	18	33
*Macvicaria maamouriae*	95.36	95.36	95.36	95.36	95.13	95.58	95.13	95.13	94.42	96.05	97.21		13	2	10	11	2	18	33
*Macvicaria gibsoni*	95.34	95.34	95.34	95.34	95.34	96.26	95.8	95.8	95.33	96.73	98.83	96.98		13	7	6	15	20	36
*Macvicaria dubia*	95.14	95.14	95.14	95.14	94.91	95.36	94.91	94.91	94.2	96.06	97.22	99.54	96.98		10	11	4	18	33
*Macvicaria bartolii*	95.12	95.12	95.12	95.12	95.35	96.5	95.81	95.81	94.87	96.97	98.83	97.67	98.37	97.68		3	12	18	32
*Macvicaria crassigula*	95.12	95.12	95.12	95.12	95.12	96.74	95.58	95.58	94.87	96.74	98.6	97.44	98.6	97.45	99.3		13	19	33
*Macvicaria obovata*	94.9	94.9	94.9	94.9	94.66	95.12	94.66	94.66	93.95	95.58	96.74	99.53	96.51	99.07	97.21	96.98		20	35
*Pseudopycnadena fischthali*	94.43	94.43	94.43	94.43	94.9	95.81	94.66	94.9	94.42	96.51	95.81	95.81	95.35	95.82	95.81	95.58	95.35		29
*Magnaosimum brooksae*	90.26	90.26	90.26	90.26	91.18	91.16	90.72	90.95	90.7	91.63	92.33	92.33	91.63	92.34	92.56	92.33	91.86	93.26	

Percentage of bases/residues that are identical

*BS -* Boknesstrand, South Africa, *CE* - Chintsa East, South Africa, *DH* - De Hoop, South Africa

**Table 7:** Nucleotide comparison of partial 28S rDNA sequences of Opistholebetinae based on a 1,250 bp-long alignment

	Number of bases/ residues that are not identical											
	*Opistholebes* cf. *amplicoelus* DH	*Opistholebes* cf. *amplicoelus* CE	*Opistholebes amplicoelus*	*Maculifer* *diodontis*	*Parallelolebes* *virilis*	*Heterolebes maculosus*	*Pseudoheterolebes diodontis*	*Pseudoheterolebes stellaglobulus*	*Macvicaria maamouriae*	*Macvicaria* *gibsoni*	*Pseudopycnadena fischthalii*	
*Opistholebes* cf. *amplicoelus* DH		0	0	14	15	17	17	17	21	24	26	
*Opistholebes* cf. *amplicoelus* CE	0		0	14	15	17	17	17	21	24	26	
*Opistholebes amplicoelus*	100	100		14	15	17	17	17	21	24	26	
*Maculifer diodontis*	98.94	98.94	98.94		14	22	11	11	34	33	31	
*Parallelolebes virilis*	98.78	98.78	98.78	98.94		23	16	16	31	28	24	
*Heterolebes maculosus*	98.62	98.62	98.62	98.29	98.13		22	22	35	38	32	
*Pseudoheterolebes diodontis*	98.62	98.62	98.62	99.19	98.70	98.21		0	35	32	28	
*Pseudoheterolebes stellaglobulus*	98.62	98.62	98.62	99.19	98.70	98.21	100		35	32	28	
*Macvicaria maamouriae*	98.22	98.22	98.21	97.19	97.36	97.02	97.02	97.02		20	32	
*Macvicaria gibsoni*	97.96	97.96	97.96	97.28	97.62	96.77	97.28	97.27	98.30		22	
*Pseudopycnadena fischthali*	97.79	97.79	97.79	97.45	97.96	97.28	97.62	97.61	97.28	98.13		
*Macvicaria obovata*	97.92	97.92	97.89	96.99	97.16	96.83	96.99	96.99	99.75	98.21	97.19	
*Macvicaria dubia*	97.71	97.71	97.70	96.94	97.28	96.68	96.85	96.85	98.56	98.47	97.53	
*Macvicaria bartolii*	97.71	97.71	97.70	97.02	97.36	96.51	97.02	97.02	98.22	99.06	97.87	
*Macvicaria crassigula*	97.52	97.52	97.48	96.91	97.24	96.43	96.91	96.91	98.13	99.40	97.96	
*Macvicaria mormyri*	97.25	97.25	97.24	96.67	96.99	96.18	96.67	96.66	97.88	99.06	97.70	
*Propycnadenoides philippinensis*	97.28	97.28	97.24	97.16	97.32	96.51	97.32	97.31	97.54	97.45	97.70	
*Peracreadium idoneum*	96.96	96.96	96.91	96.99	97.48	96.26	97.16	97.15	96.86	97.53	98.04	
*Gaevskajatrema perezi*	96.68	96.68	96.67	96.43	96.91	96.10	96.59	96.58	96.86	97.28	97.96	
*Magnaosimum brooksae*	96.00	96.00	95.94	96.30	96.83	95.45	96.34	96.34	95.76	96.51	96.60	
*Neolebouria georgiensis*	91.00	91.00	90.94	90.90	90.86	90.77	90.60	90.59	90.83	91.06	91.23	

	*Macvicaria* *obovata*	*Macvicaria* *dubia*	*Macvicaria* *bartolii*	*Macvicaria* *crassigula*	*Macvicaria* *mormyri*		*Propycnadenoides philippinensis*	*Peracreadium idoneum*	*Gaevskajatrema perezi*	*Magnaosimum brooksae*		*Neolebouria georgiensis*
*Opistholebes* cf. *amplicoelus* DH	26	27	27	31	34		34	38	41	50		108
*Opistholebes* cf. *amplicoelus* CE	26	27	27	31	34		34	38	41	50		108
*Opistholebes amplicoelus*	26	27	27	31	34		34	38	41	50		108
*Maculifer diodontis*	38	37	36	39	42		36	38	45	46		109
*Parallelolebes virilis*	35	32	31	34	37		33	31	38	39		109
*Heterolebes maculosus*	39	39	41	44	47		43	46	48	56		110
*Pseudoheterolebes diodontis*	37	37	35	38	41		33	35	42	45		112
*Pseudoheterolebes stellaglobulus*	37	37	35	38	41		33	35	42	45		112
*Macvicaria maamouriae*	3	17	21	22	25		29	37	37	50		108
*Macvicaria gibsoni*	21	18	11	7	11		30	29	32	41		105
*Pseudopycnadena fischthali*	33	29	25	24	27		27	23	24	40		103
*Macvicaria obovata*		18	22	24	27		32	40	40	52		114
*Macvicaria dubia*	98.47		22	22	23		32	36	39	48		105
*Macvicaria bartolii*	98.13	98.13		7	12		30	32	29	44		104
*Macvicaria crassigula*	98.08	98.13	99.41		10		32	34	35	48		108
*Macvicaria mormyri*	97.81	98.05	98.98	99.19			32	35	39	47		110
*Propycnadenoides philippinensis*	97.44	97.29	97.46	97.44	97.41			25	31	46		116
*Peracreadium idoneum*	96.80	96.95	97.29	97.28	97.17		98.00		33	37		110
*Gaevskajatrema perezi*	96.76	96.69	97.54	97.17	96.84		97.49	97.33		47		114
*Magnaosimum brooksae*	95.84	95.93	96.27	96.16	96.19		96.32	97.04	96.19			106
*Neolebouria georgiensis*	90.50	91.09	91.17	91.00	90.79		90.34	90.84	90.47	91.17		
	Percentage of bases/ residues that are identical											

*AUS -* Australia, *BS -* Boknesstrand, South Africa, *CE -* Chintsa East, South Africa, *DH -* De Hoop, South Africa, *WS -* Witsand, South Africa

**Fig. 5:**
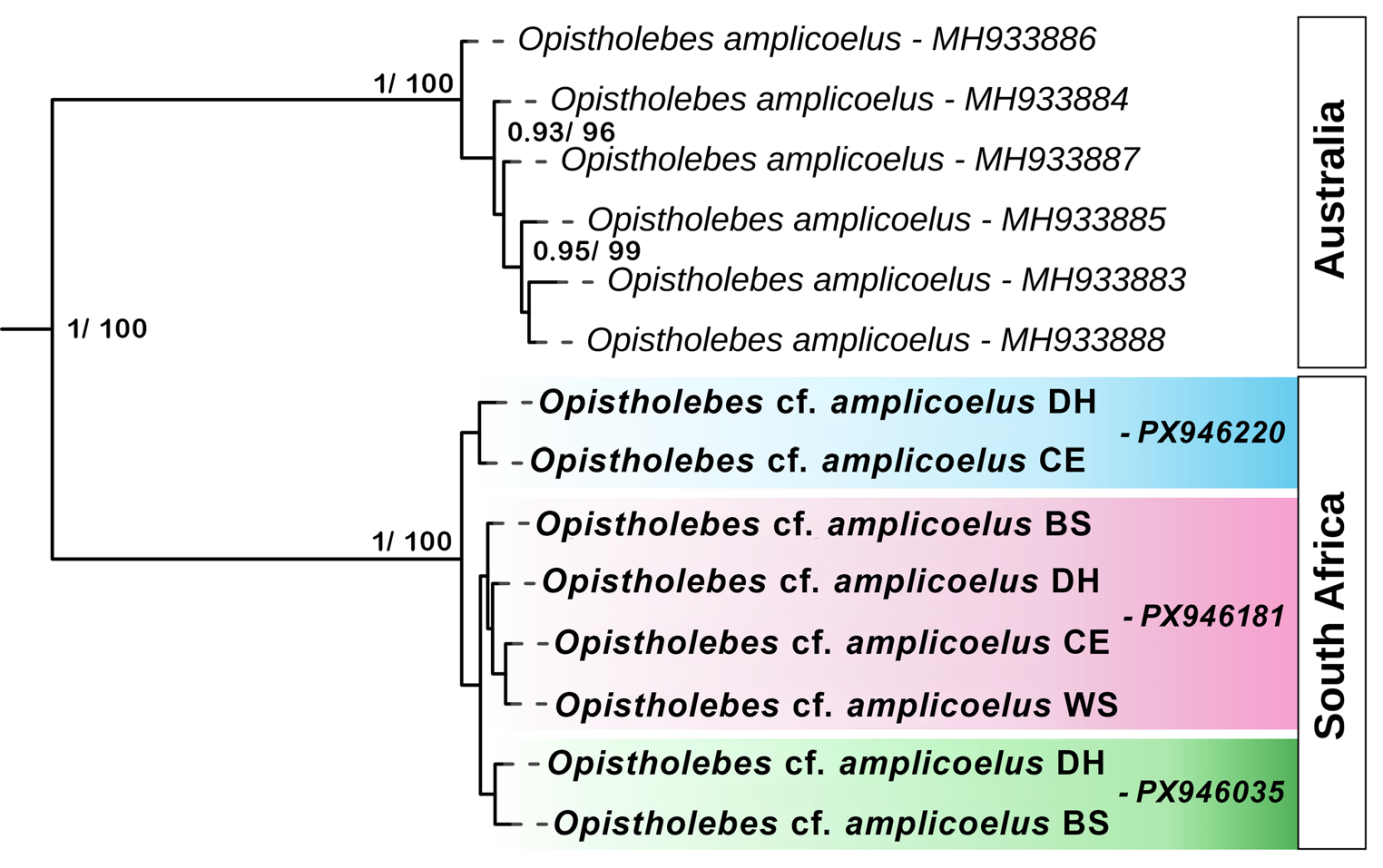
Phylogenetic tree of *Opistholebes* Nicoll 1915; trematodes based on partial *cox*1 mtDNA gene alignments. Newly generated sequences for *Opistholebes* cf. *amplicoelus* are provided in bold. Nodal support presented above or below branches for Bayesian Inference (> 0.7) and Maximum Likelihood (> 70%) analyses (BI/ML), respectively. Dashes indicate values below 0.7 and 70%. *BS* - Boknesstrand, South Africa, *CE* - Chintsa East, South Africa, *DH* - De Hoop, South Africa, *WS* - Witsand, South Africa

Alignment of the *cox*1 mtDNA dataset, comprising 11 *Opistholebes* cf. *amplicoelus* specimens from South Africa and six *O. amplicoelus* replicates from Moreton Bay, Australia, yielded 475 bp for analysis. South African specimens exhibited 0–4 bp (0–0.42%) variation at the *cox*1 mtDNA locus (Table 8), with three haplotypes identified across all localities (Fig. 5). The Australian replicates showed a comparable level of intraspecific variation (0–5 bp; 0–1.1%). However, the South African and Australian specimens differed by 69–72 bp (14.5–15.2%). Alignment of the *nad*1 mtDNA dataset yielded 474 bp for analysis. The South African specimens showed minimal variation (0–2 bp; 0–0.42%) across localities (Table 9).

**Table 8:** Nucleotide comparison of *Opistholebes* Nicoll 1915 mitochondrial *cox*1 (mtDNA) gene sequences, based on a 475 bp-long alignment

	Number of bases/residues that are not identical													
	*Opistholebes* cf. *amplicoelus* DH	*Opistholebes* cf. *amplicoelus* WS	*Opistholebes* cf. *amplicoelus* BS	*Opistholebes* cf. *amplicoelus* CE	*Opistholebes* cf. *amplicoelus* DH	*Opistholebes* cf. *amplicoelus* CE	*Opistholebes* cf. *amplicoelus* WS	*Opistholebes* cf. *amplicoelus* BS	*Opistholebes amplicoelus* AUS	*Opistholebes amplicoelus* AUS	*Opistholebes amplicoelus* AUS	*Opistholebes amplicoelus* AUS	*Opistholebes amplicoelus* AUS	*Opistholebes amplicoelus* AUS
*Opistholebes* cf. *amplicoelus* DH		0	0	0	1	1	2	2	69	71	71	71	72	72
*Opistholebes* cf. *amplicoelus* WS	100		0	0	1	1	2	2	69	71	71	71	72	72
*Opistholebes* cf. *amplicoelus* BS	100	100		0	1	1	2	2	69	71	71	71	72	72
*Opistholebes* cf. *amplicoelus* CE	100	100	100		1	1	2	2	69	71	71	71	72	72
*Opistholebes *cf. *amplicoelus* DH	99.79	99.79	99.79	99.79		2	3	1	68	70	70	70	71	71
*Opistholebes* cf. *amplicoelus *CE	99.79	99.79	99.79	99.79	99.58		1	3	68	70	70	70	71	71
* Opistholebes* cf. *amplicoelus* WS	99.58	99.58	99.58	99.58	99.37	99.79		4	69	71	71	71	72	72
*Opistholebes* cf. *amplicoelus* BS	99.58	99.58	99.58	99.58	99.79	99.37	99.16		69	71	71	71	72	72
*Opistholebes amplicoelus* AUS	85.47	85.47	85.47	85.47	85.68	85.68	85.47	85.47		2	2	4	5	3
*Opistolebes amplicoelus* AUS	85.05	85.05	85.05	85.05	85.26	85.26	85.05	85.05	99.58		0	2	3	1
*Opistholebes amplicoelus* AUS	85.05	85.05	85.05	85.05	85.26	85.26	85.05	85.05	99.58	100		2	3	1
*Opistholebes amplicoelus* AUS	85.05	85.05	85.05	85.05	85.26	85.26	85.05	85.05	99.16	99.58	99.58		3	1
*Opistholebes amplicoelus* AUS	84.84	84.84	84.84	84.84	85.05	85.05	84.84	84.84	98.95	99.37	99.37	99.37		2
*Opistholebes amplicoelus* AUS	84.84	84.84	84.84	84.84	85.05	85.05	84.84	84.84	99.37	99.79	99.79	99.79	99.58	

Percentage of bases/residues that are identical

*AUS -* Australia, *BS -* Boknesstrand, South Africa, *CE -* Chintsa East, South Africa, *DH -* De Hoop, South Africa, *WS -* Witsand, South Africa

**Table 9:** Nucleotide comparison of *Opistholebes* cf. *amplicoelus* nuclear *nad*1 (nDNA) gene sequences based on a 474 bp-long alignment

	Number of bases/residues that are not identical					
	*Opistholebes* cf. *amplicoelus* DH	*Opistholebes* cf. *amplicoelus* WS	*Opistholebes* cf. *amplicoelus* BS	*Opistholebes* cf. *amplicoelus* CE	*Opistholebes* cf. *amplicoelus* DH	*Opistholebes* cf. *amplicoelus* CE
*Opistholebes *cf. *amplicoelus* DH		0	0	0	1	1
*Opistholebes* cf. *amplicoelus* WS	100		0	0	1	1
*.Opistholebes* cf. *amplicoelus* BS	100	100		0	1	1
*Opistholebes* cf. *amplicoelus* CE	100	100	100		1	1
*Opistholebes* cf. *amplicoelus* DH	99.79	99.79	99.79	99.79		2
*Opistholebes* cf. *amplicoelus* CE	99.79	99.79	99.79	99.79	99.58	-

Percentage of bases/residues that are identical

*BS -* Boknesstrand, South Africa, *CE -* Chintsa East, South Africa, *DH -* De Hoop, South Africa, *WS -* Witsand, South Africa

## Discussion

Through detailed morphological analyses, the newly collected specimens conform closely to *O. amplicoelus* in all key characters. In particular, body proportions, sucker size ratios, gonadal arrangement, and the absence of pronounced tegumental folds around the ventral sucker all match *O. amplicoelus* as described by Nicoll (1915) and redescribed by Martin et al. (2018a). These features distinguish the specimens from *P. cotylophorus* and strongly support the identification of the South African specimens as *Opistholebes* cf. *amplicoelus*. Host associations further reinforce this taxonomic assignment. *Pseudoheterolebes cotylophorus* was described from a diodontid fish in Japan and has been reported primarily from diodontid hosts (Ozaki 1937a, b; Yamaguti 1970; Manter and Pritchard 1962). In contrast, confirmed *O. amplicoelus* records (Nicoll 1915; Martin et al. 2018a) are all from tetraodontid and related tetraodontiform fishes (pufferfishes in genera *Amblyrhynchote* Bibron, *Arothron* Müller, *Dichotomyctere* Duméril, *Lagocephalus* Swainson, and *Tetractenos* Hardy). The occurrence of *Opistholebes* cf. *amplicoelus* in *A. honckenii* off South Africa fits this host pattern and is ecologically consistent. By recognising the South African population as *Opistholebes* cf. *amplicoelus*, this study aligns the taxonomy with host–parasite ecology.

The molecular data further reinforces this conclusion. *Opistholebes amplicoelus* was originally described from tetraodontiform fish in Cleveland Bay, Australia (Nicoll 1915), and has since been infrequently reported, sometimes under different names that have been subsequently synonymised (e.g., Gupta 1968; Liu 1999). Martin et al. (2018a) provided the first modern redescription of *O. amplicoelus* from fresh material in eastern Australia, along with DNA sequences (ITS2 rDNA, 28S rDNA, and *cox*1 mtDNA). Sequences from the ribosomal markers (ITS2 and 28S rDNA) were identical across all South African specimens and to reference sequences of *O. amplicoelus* (Martin et al. 2018a), exhibiting no intraspecific variation. Phylogenetic analyses based on ITS2 and partial 28S rDNA placed these specimens in a well-supported clade with *O. amplicoelus*, distinct from *Pseudoheterolebes* spp, which formed a separate, distinct lineage. Collectively, the morphological and molecular evidence support the view that the specimen described by Bray (1986) as *P. cotylophorus* is actually *Opistholebes* cf. *amplicoelus*. Bray’s conclusion was understandable given the knowledge base and diagnostic resources available at the time. The current clarification reflects advances in modern taxonomic practice and restores *P. cotylophorus* to its known range in Japanese diodontids. Furthermore, the present study adds the first records of *O. amplicoelus* from the western Indian Ocean, expanding its known range to South Africa. It will be important for future work to include putative *O. amplicoelus* specimens from other regions (e.g. records from Asia) in molecular analyses to confirm their identity, as some of those may also prove to be misidentified taxa. Likewise, incorporating additional *Opistholebes* species (such as *O. microovus* Ku and Shen 1965) will help refine the subfamily’s systematics.

Notably, while the nuclear DNA (ITS2, 28S rDNA) of *O. amplicoelus* is uniform across the Indo-West Pacific (no observed differences between South African and Australian specimens), a substantial discordance in the mitochondrial DNA was detected. Mitochondrial *cox*1 sequences from South African vs. Australian *O. amplicoelus* differ by 68–72 bp (approximately 14–15% uncorrected divergence). In contrast, within South African representatives, the *cox*1 mtDNA sequences were very similar (only 0–4 bp or 0–0.4% variation), and the Australian sequences examined by Martin et al. (2018a) were likewise homogeneous (0–5 bp or 1.1% intraspecific *cox*1 variation).

Large intraspecific mitochondrial divergences are unusual but not unprecedented in marine trematodes (Huston et al. 2024). For context, recent studies have reported an increasing mtDNA divergence within single trematode species. For example, Magro et al. (2023) found up to 8.9% *cox*1 mtDNA divergence among specimens of *Paraschistorchis stenosoma* (Hanson, 1953) Blend Karar and Donen, 2017, in sympatry that were nonetheless considered one species. Cribb et al. (2024) documented 11.2% *cox*1 mtDNA divergence between Japanese and Australian populations of *Prodistomum orientale* (Layman, 1930) Bray and Gibson, 1990, which they treated as conspecific. Even more striking, Cutmore et al. (2023) noted ~ 12–13% mitochondrial divergence within *Transversotrema enceladi* Cutmore et al. 2023, parasites on the Great Barrier Reef. Huston et al. (2021) demonstrated that specimens of *Gorgocephalus kyphosi* Manter, 1966, and *G. yaaji* Bray & Cribb, 2005, differed by 62 bp (13%) in the *cox*1 mtDNA region between populations spanning the Tropical Indo-west Pacific region from South Africa to French Polynesia, despite only differing in ITS2 and 28S rDNA regions by 0–3 bp in both regions (for *G. kyphosi*) and 0–5 bp and 0–6 bp (for *G. yaaji*), respectively. In these cases, high mtDNA variation did not lead to taxonomic separation, either because other genes were homogeneous or morphology was indistinguishable. The ~ 15% *cox*1 mtDNA divergence observed between the South Africa and the Australian *O. amplicoelus* does represent the upper extreme of reported intraspecific variation in marine trematodes, however, existing precedents demonstrate that even double-digit mtDNA differences can occur within recognised species. This level of divergence may reflect long-term isolation between South African and Australian lineages, which might just be beginning to diverge. While genetic distance thresholds provide useful guidance, they are not absolute and must be interpreted in the context of morphology, nuclear data, and overall biological coherence, reinforcing the need for a case-by-case approach. This pattern, with a lack of nuclear divergence and morphological distinction, yet a big mitochondrial divergence between geographically separated populations, presents an interesting evolutionary scenario.

One possibility is incomplete lineage sorting following historical isolation. If the South African and Australian populations of *O. amplicoelus* were separated for a prolonged period (e.g. by geographic or oceanographic barriers), their mitochondrial genomes could accumulate substantial differences while the species remained morphologically and (largely) genetically cohesive. Subsequent secondary contact or dispersal (e.g. via host migrations or ocean currents) could then reestablish gene flow, homogenising the nuclear genome (through recombination and interbreeding) while the distinct mitochondrial lineages persist. Bray et al. (2022) documented a comparable situation, *Preptetos laguncula* Bray & Cribb, 1996 (Lepocreadiidae), in which individuals from Heron Island (off northeastern Australia) differed from conspecifics from the Gambier Archipelago (French Polynesia) by up to 54 bp (11%) in *cox*1mtDNA, but only 0–3 bp in ITS2 rDNA, suggesting that previously isolated lineages subsequently re-encountered and merged. A similar mechanism could explain the pattern in *O. amplicoelus*, where the species may have radiated in separate ocean basins (Indian Ocean vs. Pacific) or has intermittent gene flow, such that the mtDNA differences have not equilibrated. Such a re-encounter would be facilitated by this species’ apparent broad host range, having been reported from five tetraodontid species, including species of three genera in sympatry (Martin et al. 2018a).

Another potential explanation is cryptic speciation. The ~ 15% *cox*1 mtDNA divergence is large by most standards and could indicate incipient speciation, in which the South African population is an evolutionarily significant unit. Under this scenario, *O. amplicoelus* could comprise two cryptic lineages that remain morphologically similar. However, no consistent morphological or nuclear genetic differences support formal taxonomic separation. Although some measurements differ in overall range between the South African material and the Australian specimens reported by Martin et al. (2018a) (see Table 3), the PCA analyses revealed substantial overlap in morphospace (Fig. 3), and no single character, or combination of characters, provided a reliable diagnostic distinction. This interpretation is further constrained by the relatively small South African sample size compared to the Australian material. Consequently, individual specimens cannot be confidently assigned to either population based on morphology alone. While minor morphometric shifts may exist, potentially reflecting sampling effects or environmental and host-related influences, no diagnostic features distinguish the two groups. The *cox*1 mtDNA divergence is therefore acknowledged as genuine, but under a total-evidence framework, it is insufficient, in isolation, to justify the recognition of a new species and therefore we propose treating the South African species as *Opistholebes* cf. *amplicoelus*.

Interpreting potential incipient speciation is difficult when, currently, no information is available regarding the phylogeography or host connectivity of *O. amplicoelus* across its purported range. It is widely understood that limits in gene flow and host-driven specialisation are primary drivers of parasite speciation (Santacruz et al. 2020; Poulin and Bennett 2025). An additional factor to consider is host-associated divergence or spill-over events that might create mitochondrial structure without full speciation. It appears that *O. amplicoelus* has a broad host range among pufferfishes (Martin et al. 2018a). Different host species or ecotypes might harbour distinct parasite subpopulations. It is conceivable that the lineage of *O. amplicoelus* now present in *A. honckenii* off South Africa arrived via a host spill-over from another region or host species. For instance, a stray individual of a highly vagile host might have introduced the parasite to South African waters, founding a local population with a unique mtDNA signature. Huston et al. (2024) describe a similar phenomenon of spillback in Indo-Pacific trematodes, where a parasite genotype from French Polynesia consistent with *Emprostiotrema fusum* (Goto & Ozaki, 1929) appeared in small numbers in Western Pacific locations, likely via long-distance host movement. In the present study, *A. honckenii* itself is not known for long-distance dispersal (Froese and Pauly 2025), but other tetraodontids (or intermediate hosts) could facilitate rare exchanges between the western Indian Ocean and the Pacific. Such episodic dispersal could produce isolated pockets of genetic divergence (especially in fast-evolving mtDNA) without corresponding divergence in morphology or nuclear genes.

Within South Africa, the data show minimal genetic structure. All South African specimens came from *A. honckenii* collected along ~ 700 km of coastline (Western to Eastern Cape provinces). Three closely related *cox*1 haplotypes were detected in South Africa, with no clear geographic segregation: two haplotypes were widespread (each found in multiple localities spanning hundreds of kilometres), and the third was found in two sites roughly 550 km apart. Interestingly, no *Opistholebes* cf. *amplicoelus* were found in any *A. honckenii* from our two northernmost sampling sites (Uvongo Beach and Sodwana Bay, on the subtropical KwaZulu-Natal coast). This absence may be explained by oceanographic factors. The southward-flowing Agulhas Current in eastern South Africa creates biogeographic separation from the cooler southern systems (Halo and Raj 2020), potentially limiting host or intermediate host dispersal. This oceanographic barrier, and associated breaks in host or snail distribution, may limit the natural dispersal of *Opistholebes* cf. *amplicoelus* beyond the Eastern Cape region. The identity of the intermediate host(s) for *O. amplicoelus* remains unknown, but its life cycle presumably includes a molluscan host; any such host would also be subject to biogeographic constraints. Although results suggest limited natural connectivity, anthropogenic mechanisms could still facilitate long-distance or interregional movement. For example, international aquarium trade and the inadvertent transport of snail hosts in ship ballast water could represent plausible pathways for human-mediated dispersal. Future parasitological surveys north of the current range, and life-cycle studies and monitoring of potential anthropogenic vectors, may help clarify whether parasite populations are truly isolated or whether rare long-distance introductions, either natural or human-mediated, occur across broad marine regions.

This integrative taxonomic approach identifies the South African opistholebetine trematode as *Opistholebes* cf. *amplicoelus*, clarifying that earlier records from *A. honckenii* reported as *P. cotylophorus* in fact represent this species. Although pronounced mitochondrial divergence exists between South African and Australian populations, a conservative taxonomic interpretation is adopted. This decision is supported by concordant morphology, identical nuclear markers, the absence of diagnostic characters, and the limited geographic and host coverage currently available. The mitochondrial discordance is therefore interpreted as potential intraspecific structuring or incipient differentiation within a broadly distributed species, rather than definitive evidence of speciation. Species boundaries are recognised as provisional and open to reassessment as additional data emerge. Broader geographic sampling, increased specimen numbers, expanded host coverage, and the inclusion of additional genetic markers will be essential to clarify whether these lineages represent cryptic species or structured populations of a single taxon. There is growing advocacy for the use of genome-wide markers and rapidly evolving nuclear loci such as microsatellites and single-nucleotide polymorphisms (SNPs) in species delineation (Blasco-Costa 2025). Until such evidence is available, formal taxonomic splitting based on *cox*1 mtDNA alone is considered premature. Patterns observed in southern African marine parasites further emphasise the need for caution. Several recent studies suggest low population structuring and broad distributional continuity among trematodes along this coastline, although these interpretations remain tentative due to limited sampling and incomplete life-cycle data (Louvard et al. 2025; Yong et al. 2025). Substantially expanded sampling from additional regions, hosts, and parasite populations, together with multi-gene and life-history data, will be required to rigorously test models of connectivity, divergence, and host-associated structuring. Despite these uncertainties, this study provides a robust foundation by resolving a key taxonomic ambiguity and highlighting the value of integrative approaches in illuminating digenean diversity and host–parasite phylogeography in southern African marine systems.

## Data Availability

Sequence data representing all gene regions and haplotypes generated in this study have been made available on the NCBI GenBank database. Voucher specimens have been accessioned in publicly accessible museum collections. All other data are included in the manuscript.
